# Systemic testosterone induces structural, immunological, and sex steroid receptor changes in human XX skin

**DOI:** 10.1038/s41598-026-58054-4

**Published:** 2026-06-16

**Authors:** Nina Dabrosin, Annelie Abrahamsson, Gunnar Kratz

**Affiliations:** 1 Department of Biomedical and Clinical Sciences, Linköping University, Linköping, Sweden; 2 Clinical Department of Hand and Plastic Surgery, and Burns, Region Östergötland, Linköping, Sweden; 3 Clinical Department of Oncology, Region Östergötland, Linköping, Sweden; 4 Clinical Department of Hand and Plastic Surgery, and Burns, Universitetssjukhuset, 581 85 Linköping, Sweden

**Keywords:** Sex steroid receptor modulation, Dermal architecture, Immune cell infiltration, Skin remodeling, Collagen deposition, GAHT, Cell biology, Diseases, Endocrinology, Immunology, Medical research, Physiology

## Abstract

Skin-resident cells express multiple sex steroid receptors, making the cutaneous microenvironment sensitive to hormonal fluctuations. Transgender individuals assigned female at birth receiving gender-affirming hormone treatment (GAHT) with systemic testosterone often undergo several surgical procedures, underscoring the importance of understanding how GAHT influences skin biology and wound-healing capacity. This study aimed to characterize how XX (genotype) skin responds to systemic testosterone exposure. Discarded skin samples from plastic surgeries were collected from XX patients with and without testosterone treatment and analyzed for sex steroid receptor expression, immune cell markers, and structural integrity components. In testosterone-treated skin, we observed, in dermis, a 2.77-fold increase in androgen receptor-positive cells, elevated estrogen receptor α- and progesterone receptor-positive cell counts, a 43% reduction in CD45 positive immune cells, and a 2.93-fold increase in collagen density compared with untreated controls. In the epidermis, testosterone treatment resulted in reduced progesterone receptor-positive cell numbers and increased fibronectin expression. Collectively, testosterone-treated XX skin exhibited features resembling a male skin phenotype, potentially driven by altered sex steroid receptor expression, shifts in immune cell infiltration, and changes to structural organization. These findings highlight the need for further studies to elucidate the mechanisms underlying testosterone-induced skin remodeling and its implications for surgical outcomes.

## Introduction

In recent years, the role of sex hormones in skin homeostasis and wound healing has been highlighted^1,2^ although the effect on wound healing remains controversial^3–5^. Epidermal, dermal and skin-resident immune cells express sex steroid receptors^1,6–8^ and these cells work together in a tightly orchestrated team to exert their specific functions in the processes of skin homeostasis: epidermal and dermal regeneration, and neuro-endocrine and immune system regulation^9,10^. As these skin-resident cells express multiple sex steroid receptors, the skin microenvironment may be susceptible to change when the hormone milieu changes.

Transgender assigned female at birth (AFAB) people receiving gender affirming hormone treatment (GAHT) with systemic testosterone experience various desired effects including voice deepening, fat redistribution, facial and body hair growth as well as side effects such as acne and androgenic alopecia^11,12^. Another potential unwanted outcome involves surgical complications; following chest-masculinization mastectomy the patients may experience hypertrophic and poor scarring, although the evidence remains controversial^13–15^. For more than two decades at our plastic surgery clinic we have performed gender affirming chest surgeries where we have experienced differences in the skin related to testosterone treatment. These include increased dermal firmness and increased difficulties to de-epithelialize (a procedure where the epidermis is cut off from the underlying dermis) the skin during procedures. These changes are previously not very well described and possible mechanisms behind these testosterone-related alterations remain unexplored. As many transgender individuals undergo multiple surgeries involving different organ systems (i.e. mastectomy, genital reconstructive/altering surgeries, and hystero-oophorectomy) understanding how GAHT affects not only the skin but also other tissues is essential to anticipate and/or prevent wound-healing complications. Therefore, this study aimed to characterize how XX (genotype) skin responds to systemic testosterone exposure. We show significant changes in sex steroid receptor expression, in immune cell infiltration, and in the dermal structure after testosterone treatment compared to non-treated skin. Clinically these results may be used to develop clinical guidelines regarding hormone treatments pre- and postoperatively for improved surgical outcomes.

## Material and methods

### Patient material

The patients were treated according to the latest published guidelines regarding endocrinological treatment of AFAB individuals^16^. The goal of the treatment is serum testosterone levels of 10–40 nmol/L (similar to levels of cisgender men) reached by either 1000 mg of testosterone injected every 8–12 weeks or transdermal gel treatment of 50–100 mg testosterone every day. The therapy results for most individuals in cessation of menses^16^.

Otherwise discarded skin from six AFAB (XX genotype) patients undergoing chest-masculinization mastectomy who had been treated with testosterone for at least one year (XX + Testosterone (T) skin) was collected. Likewise, otherwise discarded skin from six cisgender premenopausal women going through clinically indicated plastic surgeries (XX skin) was collected as control skin. The tissue was anonymized before sampling and analyses. The skin was cut into 8 mm discs and then formalin-fixed and paraffin-embedded.

### Immunohistochemistry

Four μm sections of the skin, mounted on slides, were deparaffinized using PT Link with Dakos antigen retrieval solution, Agilent Technologies (RRID:SCR_013575), with low pH for antigen demasking. 3% hydrogen peroxide (Merck KGaA, Darmstadt, Germany, Cat# 925B) was used to block the endogenous peroxide. For staining we used the primary antibodies, mouse anti-human CD68 (Abcam Cat# ab955, RRID:AB_307338; 1:50), mouse anti-human CD45 (SouthernBiotech, Cat# 9624-01, RRID: AB_2797013, 1:200), rabbit anti-human neutrophil elastase (Abcam, Cat# 131260, 1:250), mouse anti-human mast cell tryptase (Santa Cruz Biotechnology Cat# sc-59587, RRID:AB_793510, 1:200), rabbit anti-human estrogen receptor beta (ERβ) (LSBio (LifeSpan) Cat# LS-B945, RRID:AB_2102382, 1:100), rabbit anti-human Elastin (Bioss Cat# bs-1756R, RRID:AB_10856940, 1:200) and rabbit anti-human fibronectin (Thermo Fisher Scientific Cat# PA5-29578, RRID:AB 2547054, 1:1000). HRP conjugated goat anti-rabbit (Abcam Cat# ab6721, RRID:AB 955447) and goat anti-mouse conjugated with peroxidase labelled polymer (Dako, Cat# K4000) were used as secondary antibodies. For detection we used the DAB chromogen kit from Biocare Medical (Cat# BDB2004H) and Mayers HTX, (Histolab, Cat# 01820) for counterstaining. The AR staining was done with the primary rabbit anti-human AR (Cell Marque Cat# 200R, RRID AB 2893478) using the fully automated Leica Bond-III system (Leica Biosystems, Melbourne Pty Ltd, Melbourne, Australia) by the Clinical Department of Pathology at Linköping University Hospital, which is an accredited clinical laboratory undergoing yearly quality-controlled assays of biomarkers including ERα, PGR, and AR. All assays include positive controls. For ERα and PGR we used the primary antibodies, rabbit anti-human ERα (Roche Diagnostics GmbH, Sandhofer Strasse, Mannheim, Germany, Cat# 790-4324) and rabbit anti-human PGR (Roche, Cat# 790-2223) stained with the ultraView Universal DAB Detection Kit from Roche using the BenchMark Ultra Plus system from Hematology Analyzer Cost (Saiinoiwakecho, Ukyo Ward, Kyoto, Japan) at the Clinical Department of Pathology at Linköping University Hospital, an accredited clinical laboratory as previously described. Negative control, without primary antibody, did not show any staining. Positive control for CD68 and neutrophil elastase showed staining. The manual from the Masson’s Trichrome staining kit was used for detection of all major types of collagenous fibers (abcam, Cat# ab150686). All slides were visualized using the Olympus BX43 light/fluorescence microscope, with Olympus DP74 3CCD mode camera, and CellSens imaging software version 1.15 (Olympus CellSens Software, RRID:SCR_014551), which also was used for computer-assisted quantification.

#### Epidermal evaluation

Two blinded observers evaluated staining intensity in the entire epidermis for fibronectin, AR, and ERβ from a scale of 0–3. For PGR and ERα the percentage of positive stained cells was evaluated manually in the basal stratum of the epithelial cells by two blinded observers. The positive receptor stains were mainly cytoplasmic. These stains were performed at an accredited clinical histopathology laboratory with positive controls in each run as previously described. Because of this we evaluated also the cytoplasmatic stained cells as positive. This is in coherence with previous studies, which have shown presence of cytoplasmatic ERα and ERβ in human breast cancer cells^17,18^ and cytoplasmic PGR in keratinocytes^19^. All cells were counted in three fields per section where positive cells were found in 400× magnification. To ensure a more robust evaluation computer-assisted quantification was also used to estimate percent positive staining per marked area. One section per patient was evaluated, manual epidermal evaluation XX n = 5 (15 fields) XX + T n = 6 (18 fields), computer-assisted epidermal evaluation XX n = 5 XX + T n = 6. One XX patient was excluded due to risk of false positive staining evaluation because of many melanocytes in the basal stratum.

#### Dermal cell evaluation

For the dermal cells, two blinded observers assessed skin samples for CD45, mast cell tryptase, AR, ERα, and PGR positive cells. Three hotspots per skin sample (here defined as the most densely populated area of positive cells) were selected in 200× magnification for mast cell tryptase, PGR, AR, ERα, and in 100× magnification for CD45 by the blinded observers. The cells were then counted in 400x (mast cell tryptase, PGR, AR, ERα) and 200x (CD45) times magnification. One section per patient was evaluated. PGR, AR, ERα XX n = 6 (18 hotspots) XX + T n = 6 (18 hotspots). CD45 and mast cell tryptase XX n = 5 (15 hotspots) XX + T n = 6 (18 hotspots). One XX patient was excluded due to risk of false positive staining evaluation because of many melanocytes in *stratum basale*.

#### Dermal component evaluation

Collagen and fibronectin density was quantified as percentage of positive staining per hotspot (here defined as the area with the densest and the most intense staining) selected by the blinded observers in 400× magnification using computer-assisted quantification. One section per patient was evaluated XX n = 6 XX + T n = 6.

#### Statistical analysis

Data were tested for normality and analyzed using unpaired two-tailed t-test. GraphPad Prism Version 10.6.1 (799) (GraphPad Software, LLC, Boston, MA USA) was used for statistical analyses. A *p*-value of < 0.05 was considered significant. Data are displayed as mean ± SEM. Statistical significance is displayed by ns *P* > 0.05, *P ≤ 0.05, **P ≤ 0.01, ***P ≤ 0.001, ****P ≤ 0.0001.

## Results

### Sex steroid receptors

First, we investigated the effect of testosterone on sex steroid receptor expression and stained all skin samples for androgen receptors (AR), progesterone receptor (PGR), and estrogen receptor α (ERα) and estrogen receptor β (ERβ). The distribution of the receptors was evaluated in both epidermis and dermis.

#### Androgen receptors

**Fig. 1:**
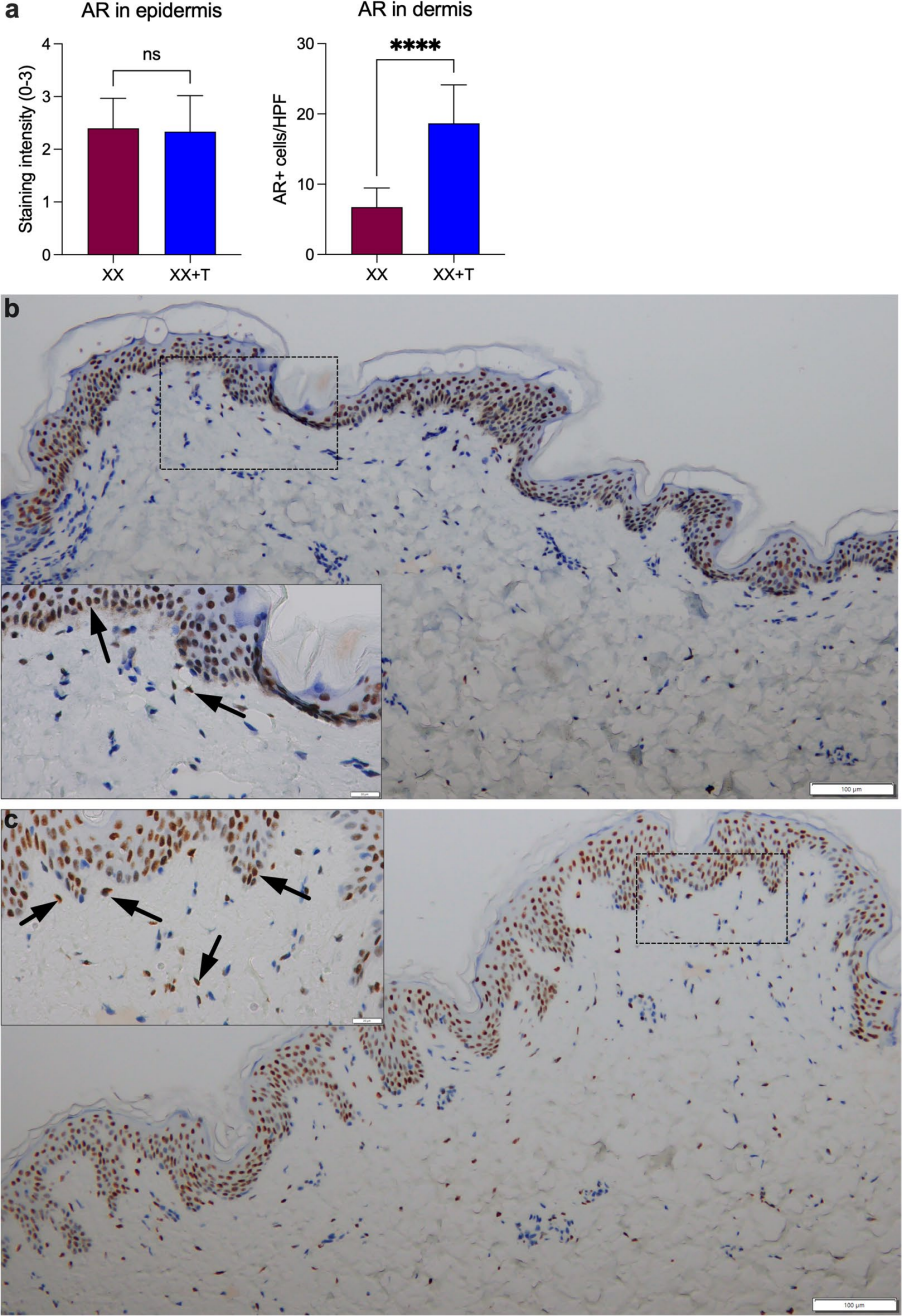
Androgen receptor (AR) expression. All skin samples were assessed for AR. Positive cells were found both in epidermis and dermis. The epidermal assessment was performed by two blinded observers grading staining intensity (0–3). In the dermis, three hotspots per biopsy were chosen and the cells were counted by two blinded observers in 400× magnification. (**a**) Bars represent mean ± SD. XX n = 6 (18 hotspots) XX + T n = 6 (18 hotspots) ns *P* > 0.05, ****P ≤ 0.0001. (**b**) AR staining of XX skin. (**c**) AR staining of XX + T skin. Arrows indicate examples of positively stained cells. Scale bar zoomed out image = 100 μm. Scale bar zoomed in image = 20μm.

We found AR expression in epidermis in all patients with no difference in intensity (XX vs. XX + T; 2.4 vs. 2.33 staining intensity, mean difference ± SEM − 0,067 ± 0,27 *P* = 0.81) (Fig. 1) or distribution. However, in dermis we found a 2.77-fold increase of AR positive cells in XX skin treated with testosterone (XX + T skin) compared to untreated control skin (XX skin) (XX vs. XX + T; 6.72 vs. 18.67 cells/hotspot, mean difference ± SEM 11.94 ± 1.44 *P* ≤ 0.0001) (Fig. 1).

#### Estrogen receptors

**Fig. 2:**
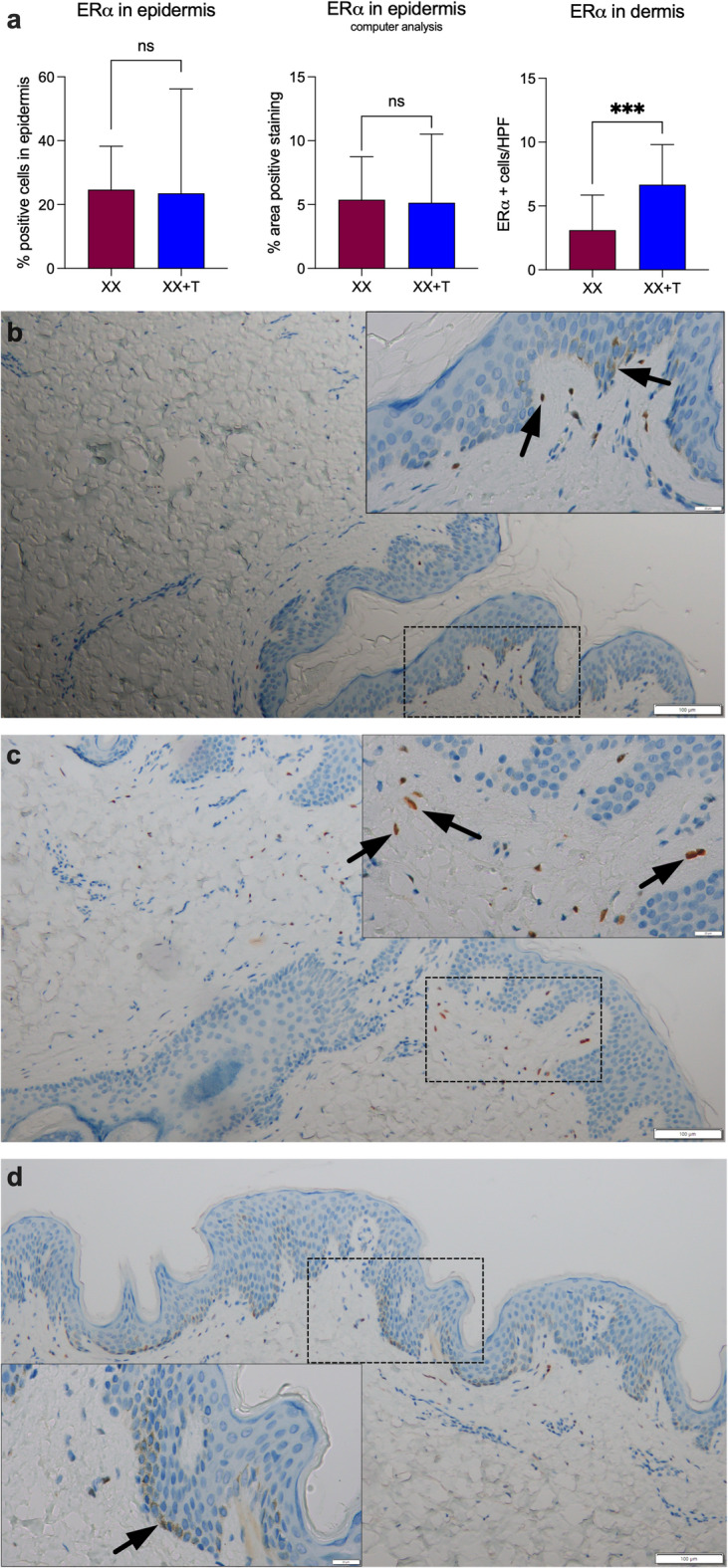
Estrogen receptor alpha (ERα) expression. All skin samples were assessed for ERα. Positive cells were found both in epidermis and dermis. In the epidermis, percentage of positive cells was assessed by two blinded observers and computer-assisted percent area of positive staining was quantified. In the dermis, three hotspots per biopsy were chosen and the cells were counted by two blinded observers in 400× magnification. (**a**) Bars represent mean ± SD. Manual epidermal evaluation XX n = 5 (15 fields) XX + T n = 6 (18 fields), computer-assisted epidermal evaluation XX n = 5 XX + T n = 6, and dermal evaluation XX n = 6 (18 hotspots) XX + T n = 6 (18 hotspots) ns *P* > 0.05, ***P ≤ 0.001. (**b**) ERα staining of XX skin representative of positive cells in epidermis and dermis. (**c**) ERα staining of XX + T skin, representative of positive cells in dermis. (**d**) ERα staining of XX + T skin, representative of positive cells in epidermis. Arrows indicate examples of positively stained cells. Scale bar zoomed out image = 100 μm. Scale bar zoomed in image = 20μm.

We found that ERα positive cells were present in both epidermis and dermis. In epidermis, we found no difference in percentage of positive cells when counted manually (XX vs. XX + T; 24.67 vs 23.52% positive cells, mean difference ± SEM − 1.15 ± 9.05 *P* = 0.90) or by computer-assisted quantification (XX vs. XX + T; 5.38 vs 5.14% area positive staining, mean difference ± SEM − 0.24 ± 2.78 *P* = 0.93), whereas an increase of ERα positive cells in the dermis of XX + T skin compared to XX skin was revealed (XX vs. XX + T; 3.11 vs. 6.57 cells/hotspot, mean difference ± SEM 3.56 ± 0.98 *P* ≤ 0.001) (Fig. 2).

**Fig. 3:**
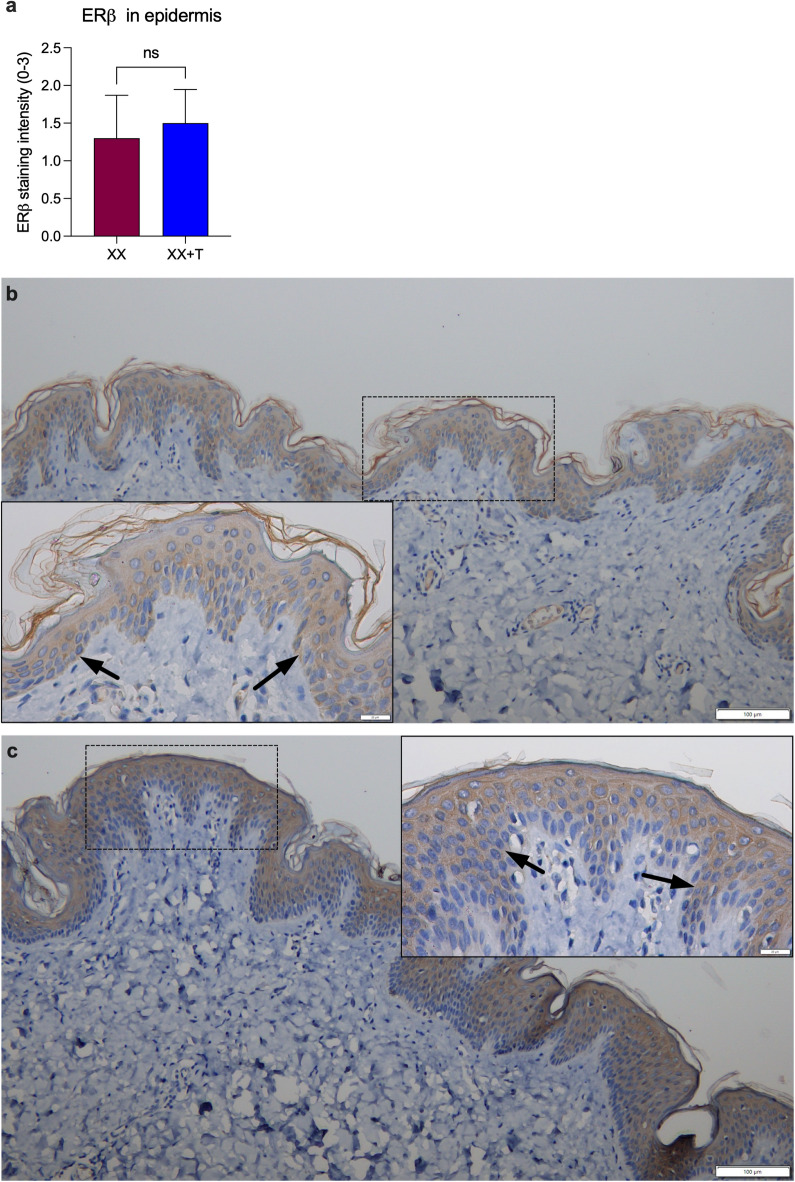
Estrogen receptor beta (ERβ) expression. All skin samples were assessed for epidermal ERβ staining intensity. Positive cells were found in the epidermal layers. Staining intensity was graded from 0 to 3 by two blinded observers. (**a**) Bars represent mean ± SD. XX n = 5 XX + T n = 6. ns p > 0.05. (**b**) ERβ staining of XX skin. (**c**) ERβ staining of XX + T skin. Arrows indicate examples of positively stained cells. Scale bar zoomed out image = 100 μm. Scale bar zoomed in image = 20μm.

ERβ positive cells were present in the epidermal layers in all skin samples, with no difference in staining intensity between the two groups (XX vs. XX + T; 1.3 vs. 1.5 staining intensity, mean difference ± SEM 0,20 ± 0,31 *P* = 0.53) (Fig. 3).

#### Progesterone receptor

**Fig. 4:**
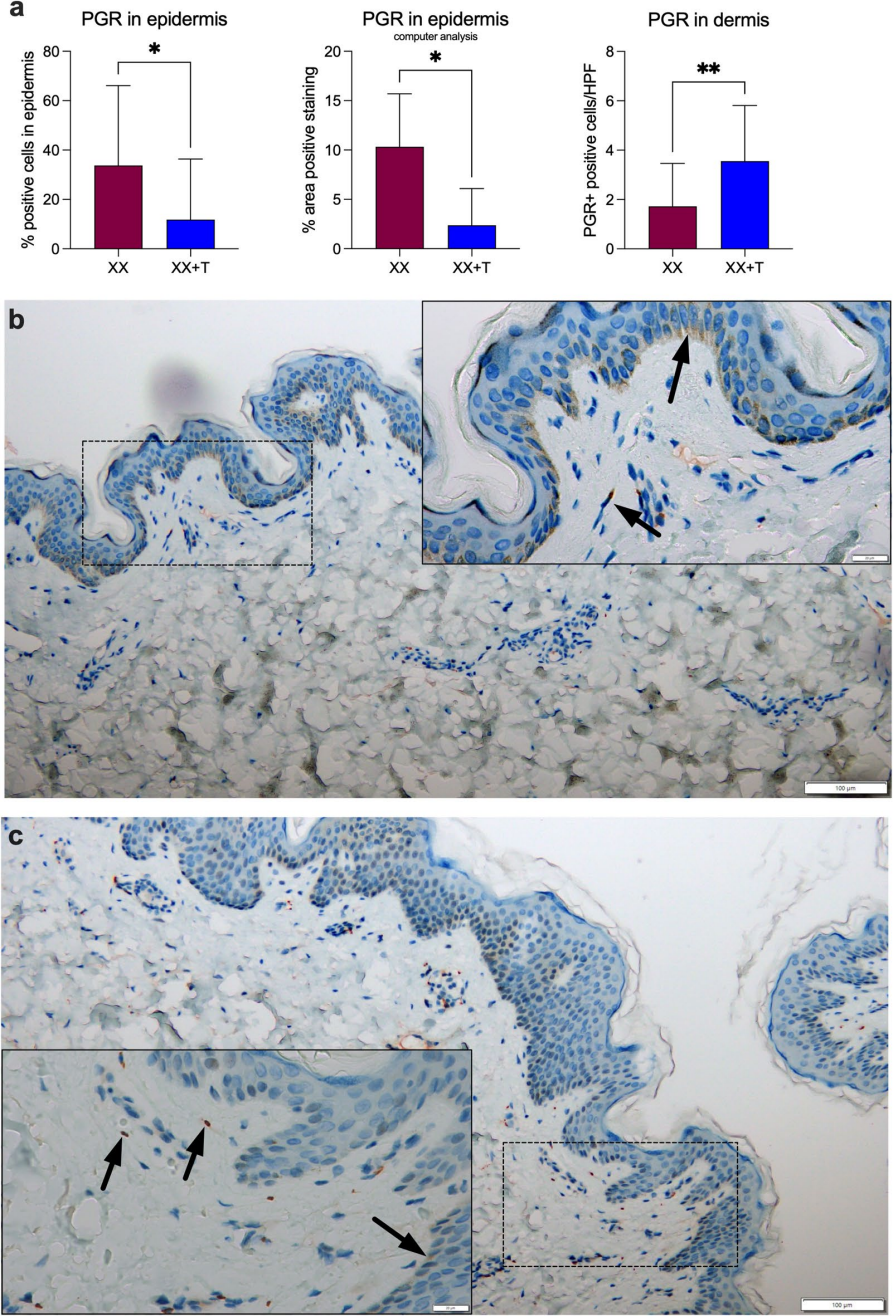
Progesterone receptor (PGR) expression. All skin samples were assessed for PGR. Positive cells were found both in epidermis and dermis. In the epidermis, percentage of positive cells was assessed by two blinded observers and computer-assisted percent area of positive staining was quantified. In the dermis, three hotspots per biopsy were chosen and the cells were counted by two blinded observers in 400× magnification. (**a**) Manual epidermal evaluation XX n = 5 (15 fields) XX + T n = 6 (18 fields), computer-assisted epidermal evaluation XX n = 5 XX + T n = 6, and dermal evaluation XX n = 6 (18 hotspots) XX + T n = 6 (18 hotspots) *P ≤ 0.05, **P ≤ 0.01. (**b**) PGR staining of XX skin. (**c**) PGR staining of XX + T skin. Arrows indicate examples of positively stained cells. Scale bar zoomed out image = 100 μm. Scale bar zoomed in image = 20μm.

We observed PGR in both epidermis and dermis. In epidermis, PGR was expressed in the basal stratum and in the dermis PGR expression was scattered. We found a significant difference in percent positive cells in the epidermis where the receptor was downregulated in XX + T skin when quantified manually (XX vs. XX + T; 33.78 vs 11.85% positive cells, mean difference ± SEM − 21.93 ± 9.90 *P* = 0.03) and by using computer-assisted quantification (XX vs. XX + T; 10.32 vs 2.38% area positive staining, mean difference ± SEM − 7.94 ± 1.75 *P* = 0.02), whereas in the dermis there was a significant upregulation of PGR in XX + T skin compared to XX skin (XX vs. XX + T; 1.72 vs. 3.56 cells/hotspot, mean difference ± SEM 1.83 ± 0.67 *P* ≤ 0.01) (Fig. 4).

### Immune cells

**Fig. 5:**
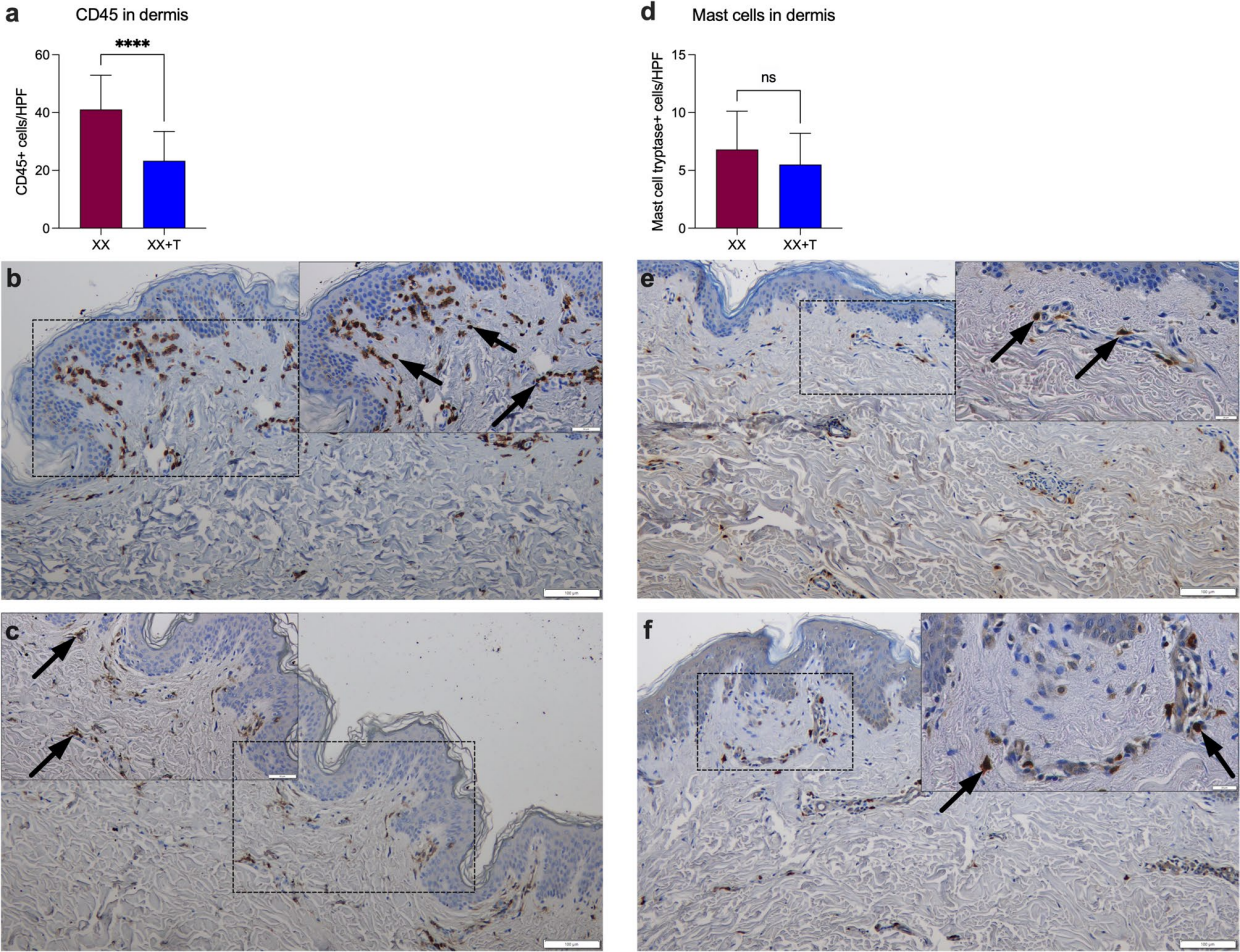
CD45 and mast cell tryptase expression. All skin samples were assessed for CD45 and mast cell tryptase. Positive cells were mainly found in the dermis. Three hotspots per biopsy were chosen and the cells were counted by two blinded observers in 200x (CD45) and 400x (mast cell tryptase) magnification. (**a**,  **d**) Bars represent mean ± SD. XX n = 5 (15 hotspots) XX + T n = 6 (18 hotspots) ns *P* > 0.05, ****P ≤ 0.0001. (**b**) CD45 staining of XX skin. (**c**) CD45 staining of XX + T skin. Scale bar zoomed out image = 100 μm. Scale bar zoomed in image = 50μm. (**e**) Mast cell tryptase staining of XX skin (**f**) Mast cell tryptase staining of XX + T skin. Scale bar zoomed out image = 100 μm. Scale bar zoomed in image = 20μm. Arrows indicate examples of positively stained cells.

**Fig. 6:**
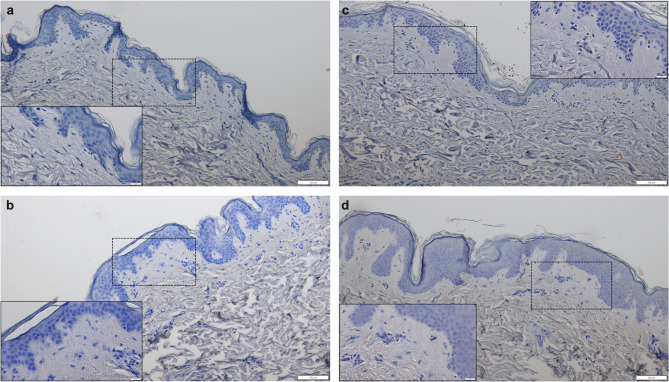
CD68 and neutrophil elastase expressions. All skin samples were assessed for both stains and no positive cells were found. (**a**) CD68 staining of XX skin (**b**) CD68 staining of XX + T skin (**c**) Neutrophil elastase staining of XX skin (**d**) Neutrophil elastase staining of XX + T skin. Scale bar zoomed out image = 100 μm. Scale bar zoomed in image = 20μm.

Next, we evaluated the immune cell infiltration in the skin samples by staining for CD45, CD68, neutrophil elastase, and mast cell tryptase. In addition to staining for a pan-leukocyte marker (CD45), macrophages (CD68) and neutrophils (neutrophil elastase), we chose to include mast cells (mast cell tryptase) in the evaluations as these cells are also a part of the innate immune response. Furthermore, mast cells are a skin-resident immune cell population^20^ and have been shown to be responders to estrogens, progesterone and testosterone^6,21^. CD45 positive immune cells were mainly found scattered in the dermis. We found a significant down regulation by 43% for CD45 + immune cells in XX + T skin compared to XX skin (XX vs. XX + T; 41.07 vs. 23.33 cells/hotspot mean difference ± SEM 17.73 ± 3.83 *P* ≤ 0.0001) (Fig. 5). Tryptase positive mast cells were mainly located around vessels in the dermis, but we observed no difference between mast cell numbers in the two groups (XX vs. XX + T; 6.80 vs. 5.50 cells/hotspot mean difference ± SEM 1.30 ± 1.049 *P* = 0.22) (Fig. 5). For CD68 and neutrophil elastase no positive cells were found (Fig. 6).

### Skin structure and integrity

**Fig. 7:**
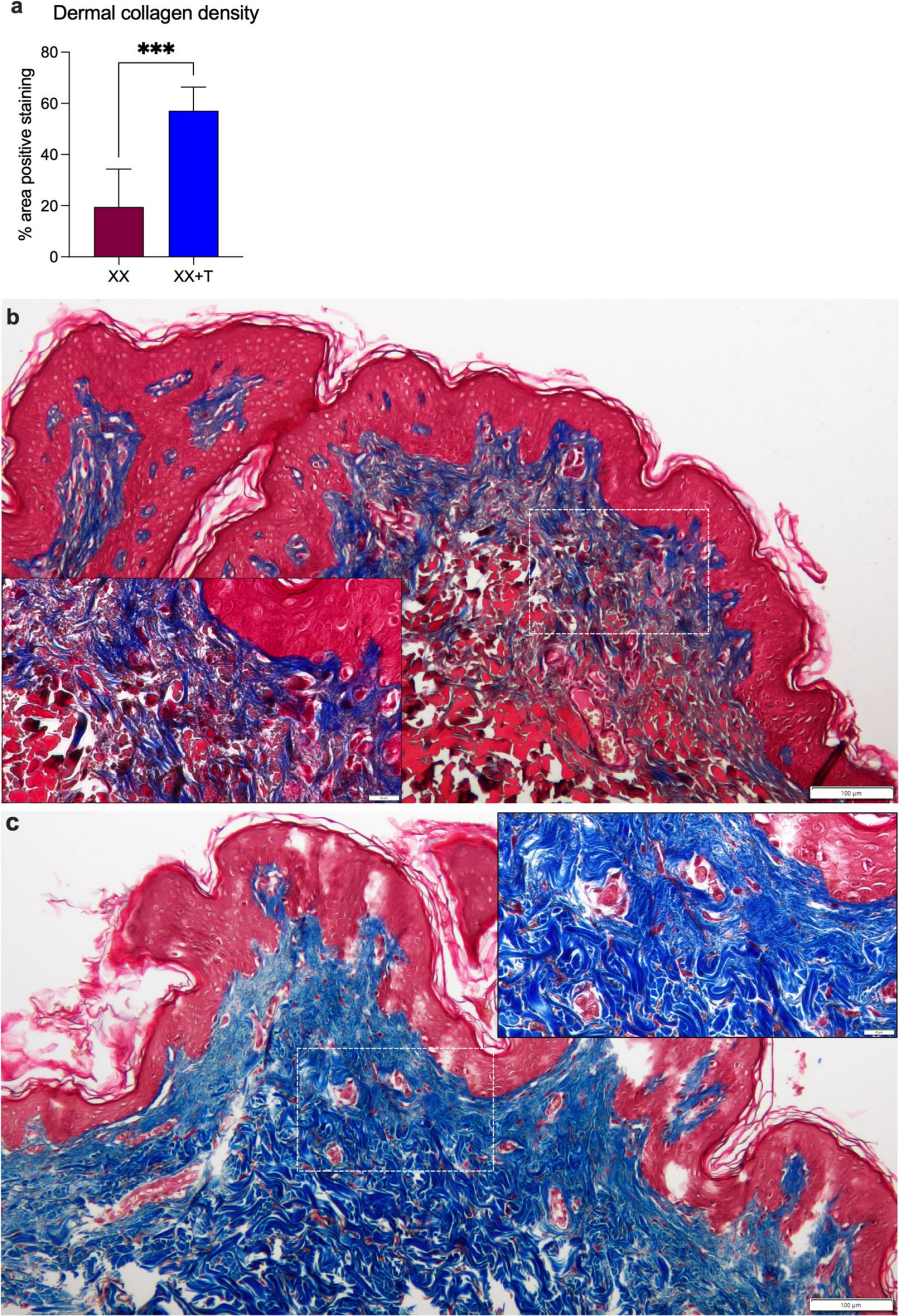
Collagen density. All skin samples were assessed for collagen density. Blue stain indicates positive staining for collagen. A hotspot was chosen in 400X magnification just below the *stratum basale* and percent area of positive staining was quantified by computer software. (**a**) Bars represent mean ± SD. XX n = 6 XX + T n = 6. ***P ≤ 0.001. (**b**) Masson’s trichrome stain of XX skin. (**c**) Masson’s trichrome stain of XX + T skin. Scale bar zoomed out image = 100 μm. Scale bar zoomed in image = 20μm.

**Fig. 8:**
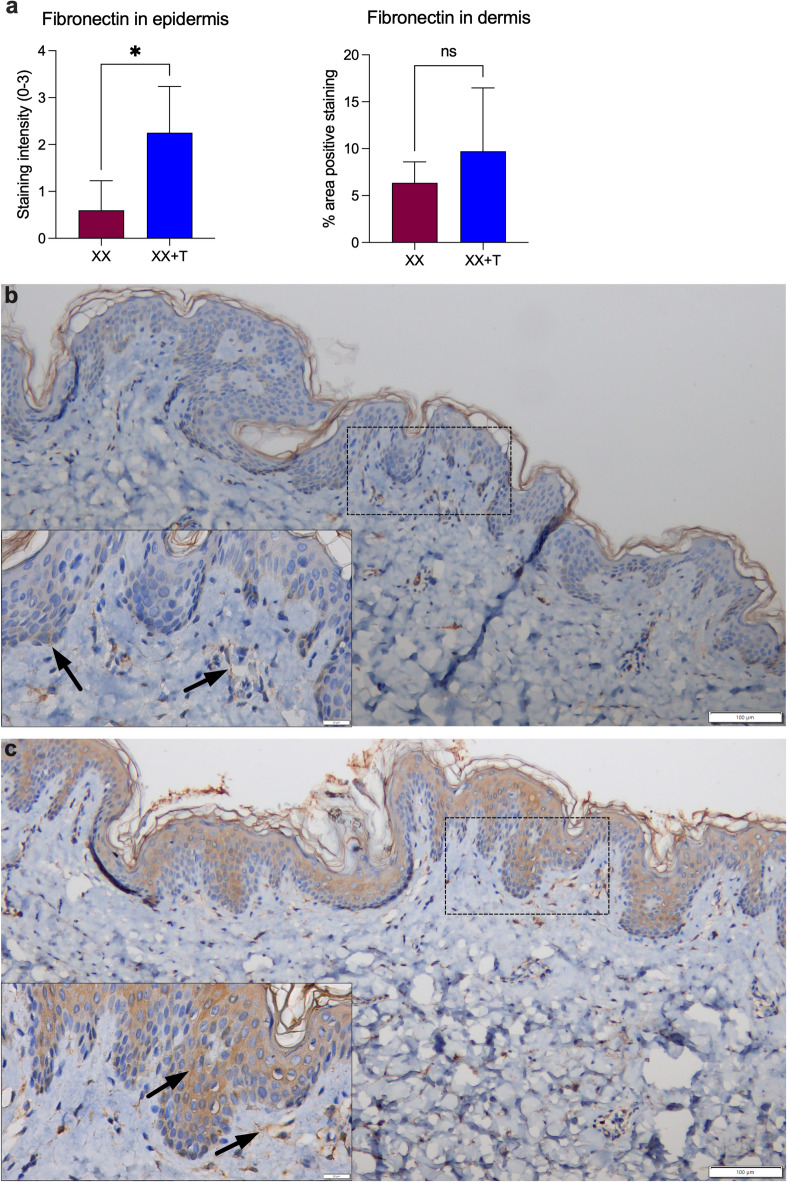
Fibronectin expression. All skin samples were assessed for fibronectin. In the epidermis, staining intensity was evaluated from 0 to 3 by two blinded observers. In the dermis, a hotspot was chosen in 400X magnification just below the *stratum basale* and percent area of positive staining was quantified by computer software. (**a**) Bars represent mean ± SD. Epidermal evaluation XX n = 5 XX + T n = 6 and dermal evaluation XX n = 6 XX + T n = 6. ns *P* > 0.05, *P ≤ 0.05. (**b**) Fibronectin staining of XX skin. (**c**) Fibronectin staining of XX + T skin. Arrows indicate examples positive staining. Scale bar zoomed out image = 100 μm. Scale bar zoomed in image = 20μm.

Lastly, we sought to assess the effects of testosterone on the structure and integrity of the skin. We stained for collagen, fibronectin, and elastin. We found a 2.93-fold increase of collagen density in the dermal layers for XX + T skin compared to XX skin (XX vs. XX + T; 19.49 vs. 57.12% area positive staining, mean difference ± SEM 37.63 ± 7.13 *P* = 0.0004) (Fig. 7). Surprisingly, fibronectin expression was found both in epidermis and dermis in both groups. We found that XX + T skin had a stronger expression of fibronectin in epidermis (XX vs. XX + T; 0.60 vs. 2.25 staining intensity, mean difference ± SEM 1.65 ± 0.51 *P* = 0.0105), but there was no difference in the fibronectin density in the dermis (XX vs. XX + T; 6.35 vs. 9.72% area positive staining, mean difference ± SEM 3.37 ± 2.90 *P* = 0.27) (Fig. 8). For elastin, we found no difference of the protein expression in the two groups.

## Discussion

Here we show that GAHT with testosterone induces profound changes in the skin. Modifications of sex steroid receptor expression in epidermis and dermis, immune cell infiltration, and structural integrity of the skin were revealed. Major effects of testosterone in the dermis were found with an increased number of AR + , ERα + , and PGR + cells. Whereas the effects in sex steroid receptor expression in epidermis were less pronounced. Additionally, CD45 + immune cells were decreased by 43% and there was an almost threefold increase of collagen in the dermis. In the epidermis a decrease of PGR + cells and an increase in fibronectin expression was revealed.

As previously reviewed, the greatest dissimilarity between male and female skin is related to differences in the dermis^22^, which may be related to physiological differences in sex hormone levels. The skin changes phenotypically when the hormone levels change during a lifetime (i.e. during menopause) or during exogenous hormone treatment^2^. Generally, in estrogen-deficient skin, collagen deposition is slower, the elasticity is poorer, and dryness is increased due to lowered sebum production and altered levels of hyaluronic acids^23^ properties partly due to decreased fibroblast proliferation, collagen deposition and increased release of matrix metalloproteinases^24^. The role of progesterone is less studied, but it has been reported that peri- and postmenopausal women using progesterone cream display improved mechanical viscoelastic properties in the skin^25^. Progesterone has also been shown to increase fibroblasts’ collagen deposition^26^. Androgens exert effects on multiple skin cells in skin homeostasis. Testosterone and 5α-dihydrotestosterone increase sebocyte proliferation^27^ and in the dermal papilla, androgens alter the gene expression related to hair growth^28^, the effects however, on keratinocytes seems to be controversial as recently reviewed^29^. These previously described effects of sex steroids in cis skin might be a consequence of alterations of sex steroid receptor expression during different hormonal stages in life. Thus, our data support that the XX phenotype of the skin is more dependent on the hormonal milieu rather than the genetic composition. Interestingly, the skin may not only work as a target organ for sex hormones as described, but may also act as a peripheral endocrine organ and is therefore involved in processes related to sex steroid synthesis, conversion and metabolization^1,29,30^.

Skin-resident immune cells consist of both myeloid and lymphoid cells, and the majority of these cells express both the AR and ER^20,21,31–33^. Androgens seem to have a general suppressive effect on the immune system regulation and inflammation^31^. Likewise, progesterone promotes an anti-inflammatory effect on the immune system^34^. Whereas estrogens can exert both anti- and proinflammatory signaling dependent on context and hormonal concentration^35^. A recent study provided evidence that transgender men under testosterone treatment acquired a male immunophenotype. Testosterone was found to regulate the signaling axis between type-I interferon and tumor necrosis factor similar to a cisgender male immune response^36^. In our study, we demonstrate that testosterone treatment exerts not only systemic immunomodulatory effects but also alters the local immune landscape of the skin. Specifically, we observed a reduction of CD45 + cells in the dermis following therapy. CD45 is a pan-leucocyte marker for almost all differentiated hematopoietic cells^37^, suggesting that testosterone broadly limits immune cell presence in the tissue. We did not find any cells expressing CD68, mainly a monocyte/macrophage marker^38^, or neutrophils and no changes in mast cell content between the groups. In line with an immunosuppressive effect of testosterone in our study, a murine model displayed that androgens dampen cutaneous immune responses^39^. Together, these findings suggest that testosterone reduces immune surveillance within the skin. Future studies will have to determine which subset of leucocytes is affected by testosterone therapy and the consequences this immunomodulation has on skin wound healing and skin autoimmunity.

Previous studies suggest that the collagen content in un-wounded male skin is higher in all ages compared to cisgender female skin^40^. Similarly, post-wounded skin from cisgender males display a higher mRNA expression of collagen and elastin than cisgender female post-wounded skin^41^. Skin collagen fibers are synthesized by dermal fibroblasts that also synthesize elastic fibers and regulate the extracellular matrix (ECM) remodeling and repair in skin homeostasis^42^. Fibroblasts express AR^43^ and in murine skin androgens have minor effects on fibroblast proliferation and migration, but a positive effect on collagen synthesis in vitro^3,44^. We found an increased accumulation in vivo of dermal AR + cells. As the most abundant cell in the dermis is the fibroblast we hypothesize that these cells may be fibroblasts. Due to the increased number of cells expressing the androgen receptor the collagen synthesis potential is augmented due to higher number of target cells for testosterone. These findings together with our finding that collagen density was significantly increased in testosterone treated skin suggest that the skin similar to the immune system of trans men acquire a male phenotype.

Fibronectin is a glycoprotein predominantly localized within the dermis, where it provides structural support for other extracellular matrix components and serves as an essential scaffold for re-epithelialization during wound repair^45,46^. Consistent with its role in regulating tissue remodeling, prior work in murine wound-healing models has shown that androgens suppress fibronectin deposition within the wound bed^44^. However, in normal, unwounded skin, the function of fibronectin remains incompletely defined. Our data reveal that fibronectin is present in both the dermal and epidermal compartments in vivo, and notably, that testosterone exposure leads to an increase in epidermal fibronectin deposition. This androgen-dependent upregulation suggests that fibronectin may have previously unrecognized roles in epidermal architecture. Moreover, elevated epidermal fibronectin and augmented dermal collagen density may help explain the increased difficulty in achieving efficient surgical de-epithelialization clinically for patients under testosterone treatment, potentially through altered cell–matrix adhesion or ECM organization. Further studies are necessary to determine the mechanistic basis of this response and its implications for surgical outcomes.

While our study offers valuable insights into hormonal skin changes it is not without limitations. These include the generalizability of our study limited by the small sample size of patients, the lack of exact testosterone dosage and duration of treatment, and the descriptive nature of our results. Our results do, however, provide a foundation for future mechanistic studies.

In conclusion, sex steroids are an essential part of skin homeostasis and exert changes depending on the systemic concentrations. In this study, we show that testosterone-treated XX-skin mimics a male skin phenotype that may be driven by changes in sex steroid receptor expression, immune cell infiltration and structural integrity in line with existing evidence.

Future studies are warranted to investigate the mechanisms behind the testosterone induced skin changes and the potential implications it has for wound healing and skin-related diseases.

## Data Availability

Data supporting the findings of this study are available from the corresponding author upon reasonable request.

## Abbreviations

AFAB: Assigned female at birth
GAHT: Gender affirming hormone treatment
ER: Estrogen receptor
PGR: Progesterone receptor
AR: Androgen receptor
XX: Untreated skin from ciswomen
XX + T: Testosterone treated skin from AFAB individuals with gender dysphoria
ECM: Extracellular matrix

## Declarations

### Generative AI and AI-assisted technologies in the writing process

During the preparation of this work the authors used ChatGPT for minor language revisions for written sentences to improve readability and language, using the following prompts: “please revise this sentence”. After using this tool, the authors reviewed and edited the content as needed and take full responsibility for the content of the publication.
